# Structural and immunogenic characterisation of ChAdOx1 Chik-derived Chikungunya VLPs supports a mechanistic rationale for vaccine-induced protection

**DOI:** 10.1038/s44298-026-00207-3

**Published:** 2026-06-30

**Authors:** Young Chan Kim, Xiyong Song, Yasunori Watanabe, Raquel de Oliveira Souza, Robert Stass, Sthefany Pagliari, Max Crispin, Beate Mareike Kümmerer, Carla Claser, Thomas A. Bowden, Juha T. Huiskonen, Arturo Reyes-Sandoval

**Affiliations:** 1 Department of Paediatrics, Oxford Vaccine Group, University of Oxford, Oxford, UK; 2 Division of Structural Biology, Centre for Human Genetics, University of Oxford, Oxford, UK; 3 Institute of Biotechnology, Helsinki Institute of Life Science HiLIFE, University of Helsinki, Helsinki, Finland; 4 Department of Parasitology, Institute of Biomedical Sciences, University of São Paulo, São Paulo, Brazil; 5 School of Biological Sciences, University of Southampton, Southampton, UK; 6 Institute of Virology, Medical Faculty, University of Bonn, Bonn, Germany; 7 German Centre for Infection Research (DZIF), Partner Site-Bonn-Cologne, Bonn, Germany; 8 Department of Microbiology, Immunology and Parasitology, Federal University of São Paulo (UNIFESP), São Paulo, Brazil; 9 Instituto Politécnico Nacional (IPN). Unidad Adolfo López Mateos, Mexico City, Mexico

**Keywords:** Immunology, Microbiology

## Abstract

Chikungunya virus (CHIKV) is a mosquito-borne alphavirus that causes severe chronic arthritis in humans and constitutes an expanding global threat. We previously reported that a single unadjuvanted dose of ChAdOx1 Chik vaccine was safe and elicited cross-protective functional antibodies against four distinctive CHIKV lineages in a Phase 1 trial. Here, we show that the CHIKV structural polyprotein encoded by ChAdOx1 Chik can assemble into CHIKV virus-like particles (VLPs) in mammalian cells. Cryo-EM derived reconstruction confirms that these CHIKV VLPs closely resemble the structure of the native virion and LC-MS demonstrates authentic E1/E2 glycosylation. In C57BL/6 mice, unadjuvanted CHIKV VLPs conferred significant protection against viremia and CHIKV-induced foot swelling. A head-to-head comparison of three adjuvant-free platforms encoding structural antigens derived from ChAdOx1 Chik showed that viral-vectors and VLPs induced similar CHIKV-neutralising antibody titres, both surpassing immunisation with soluble E2. Together, these structural and immunogenicity data support a mechanistic rationale that VLP assembly mediated by the clinically relevant ChAdOx1 Chik vaccine contributes to its vaccine-induced protection demonstrated in pre-clinical and clinical studies.

## Introduction

Chikungunya virus (CHIKV) is a mosquito-borne alphavirus that causes severe chronic arthritis in humans and has the potential to pose a risk in countries where *Aedes spp* mosquitoes are present^1,2^. Since its first identification in Tanzania in 1952, CHIKV has spread across over 100 countries in Asia, Africa, Europe and the Americas^1,3–6^. Increased outbreaks since 2004 are linked to viral adaptions that increase transmissions via *Aedes aegypti* and *Aedes albopictus*, which also spread dengue virus (DENV) and Zika virus (ZIKV)^2,5,7^. CHIKV has a single-stranded-RNA genome of ~12 kb, which encodes two polyproteins: four non-structural and five structural (s) proteins^8,9^. The non-structural proteins (nsP1, nsP2, nsP3 and nsP4) are required for virus replication^10^. The structural polyproteins (C-E3-E2-6k-E1) are processed by the capsid auto-proteinase and host signalases to release capsid C, E3, E2, 6k and E1^11^. CHIKV is enveloped with a diameter of ~ 700 Å^12^. The viral surface is mainly composed of E1-E2 heterodimers forming spikes^13^, where E1 mediates membrane fusion^14^ and E2 interacts with the host receptor, MXRA8^15,16^, and initiates clathrin-dependent endocytosis^17,18^. The E3 glycoprotein ensures the correct folding of p62, the precursor to E2, and the formation of the p62-E1 heterodimer^19,20^. E3 is cleaved by furin-like host proteases during the maturation step in the trans-Golgi network^21^.

Recently, the U.S. Food and Drug Administration (FDA) has approved two CHIKV vaccines: (1) IXCHIQ, a live-attenuated vaccine, was approved in November 2023 for individual 18 years and older at risk of exposure: (2) VIMKUNYA, a virus-like particle vaccine (VLP), was approved in February 2025 for individuals 12 years and older^22^. However, the FDA and CDC (Centers for Disease Control and Prevention) recently recommended pause in use of IXCHIQ in individuals 60 years of age or older while the investigation of post-marketing reports of serious adverse events are completed^23^. A VLP vaccine such as VIMKUNYA is considered safe due to it lacking replicating viral genetic material and is a preferred option for pregnant women^22^. VIMKUNYA contains the adjuvant aluminium hydroxide, which is widely regarded as safe and effective in enhancing vaccine immunogenicity^24,25^. Regulatory evaluations and the majority of clinical studies support the favourable safety profile of aluminium-containing adjuvants. However, a small number of observational or limited studies have raised questions regarding possible associations with neurotoxicity, macrophagic myofasciitis (MMF) or persistent asthma, although these findings remain inconclusive and require further investigation^26–30^. This highlights the value of developing next-generation vaccines that can address potential contraindications to certain platforms such as live-attenuated vaccines in pregnant or immunocompromised individuals and help overcome supply limitations of inactivated or VLP vaccines during the epidemics^31–33^.

We previously reported development of chimpanzee adenovirus virus (ChAdOx1) and Modified Vaccinia Ankara (MVA)-based viral-vectored CHIKV vaccine candidates encoding the CHIKV’s structural genes of a mosaic consensus sequence derived from multiple CHIKV lineages (Asian, ECSA, and West African). A single unadjuvanted dose of ChAdOx1 sCHIKV vaccine induced high antibody titres which were maintained even at 10 months and MVA sCHIKV was shown to be effective booster vaccine^34^. A single dose of a ChAdOx1 sCHIKV also induced high titres of neutralising antibodies and provided complete protection against a lethal CHIKV virus challenge and prevented CHIKV-associated severe inflammation^35,36^. Based on this data, the safety and immunogenicity of ChAdOx1 Chik (ChAdOx1 sCHIKV) were evaluated in a dose-escalation Phase I clinical trial (NCT03590392) in healthy adults, in which ChAdOx1 Chik induced broadly neutralising antibodies against four CHIKV lineages (Indian Ocean, West African, Asian, and Asian-American) in all participants and as early as 2 weeks after a single unadjuvanted dose^37^. Moreover, ChAdOx1 Chik induced a strong CD4^+^- IFN-γ^+^ biased cellular response toward CHIKV E1 and E2 glycoproteins but had no significant effect on CD8^+^ T cells^37^. As ChAdOx1 Chik vaccine showed excellent safety, tolerability and 100% PRNT_50_ seroconversion after a single unadjuvanted dose in Phase I clinical trial, ChAdOx1 Chik then entered a Phase 1b clinical trial (NCT04440774) in order to evaluate its safety and immunogenicity when administered alone or in co-administration with ChAdOx1-Zika in healthy adults in Mexico^38^. Based on prior observations that expression of the CHIKV structural polyprotein can drive VLP formation, including our previous work showing that HEK293 cells expressing sCHIKV secrete ~70 nm particles resembling native CHIKV virions, we hypothesised that our clinically relevant ChAdOx1 Chik vaccine would similarly direct host cells to secrete the encoded structural polyprotein in the form of VLPs that closely mimic authentic CHIKV virions, thereby presenting both linear and conformational epitopes required for potent antibody-mediated protection. Despite recent progress in CHIKV vaccines, few head-to-head comparisons of adjuvant-free CHIKV vaccine platforms have been reported.

Here, we demonstrate that the vaccine antigen in our clinically relevant ChAdOx1 Chik indeed produces CHIKV VLPs in mammalian cells. A cryo-electron microscopy-derived reconstruction confirmed that CHIKV VLPs closely resemble the structure of the native virion and LC-MS demonstrated authentic E1/E2 glycosylation. In mice, one or two unadjuvanted doses of our CHIKV VLPs significantly reduced viremia and CHIKV-induced foot swelling. Given the differing strengths and limitations of major CHIKV vaccine platforms, including rapid and scalable manufacturing of viral-vectors, the safety and strong humoral responses typically elicited by VLPs and protein sub-unit vaccines, we sought to compare these three adjuvant-free approaches. Understanding how these platforms differ in immunogenicity is important for informing vaccine design decisions and optimising deployment strategies in outbreak settings. We therefore compared the three adjuvant-free CHIKV platforms (viral vectors (ChAdOx1/MVA), VLP and protein subunits) expressing the equivalent vaccine antigen in mice. All vaccine platforms elicited high anti-E1 and anti-E2 IgG titres, however, only the viral-vectored and VLP vaccines induced neutralising antibody titres, both surpassing the protein subunit. Poly (I:C) adjuvant enhanced neutralising titres after a single VLP dose, but additional adjuvanted boosts conferred no further benefits, indicating use of adjuvants may not be needed in multi-dose CHIKV VLP vaccination regimens. Unexpectedly, mice sera collected 4 weeks after prime-boost-boost (PBB) VLP vaccination provided limited cross-neutralisation against the related arthritogenic Mayaro virus (MAYV). In summary, our clinically relevant ChAdOx1 Chik acts as a self-assembling VLP vaccine that combines the advantages of leading VLP platforms with the intrinsic immunostimulatory and T-cell priming properties of adenoviral vectors, supporting a mechanistic rationale for its vaccine-induced protection observed in pre-clinical and clinical studies. Its ability to induce robust neutralising antibody responses without the use of aluminium adjuvant may help address public concerns around cumulative aluminium exposure in childhood immunisation schedules. Together with recent concerns regarding the use of live-attenuated CHIKV vaccines, ChAdOx1 Chik could offer a promising alternative approach for CHIKV vaccination during outbreak settings.

## Results

### Clinically relevant ChAdOx1 Chik self-assembles into virus-like particles in mammalian cells

**Fig. 1:**
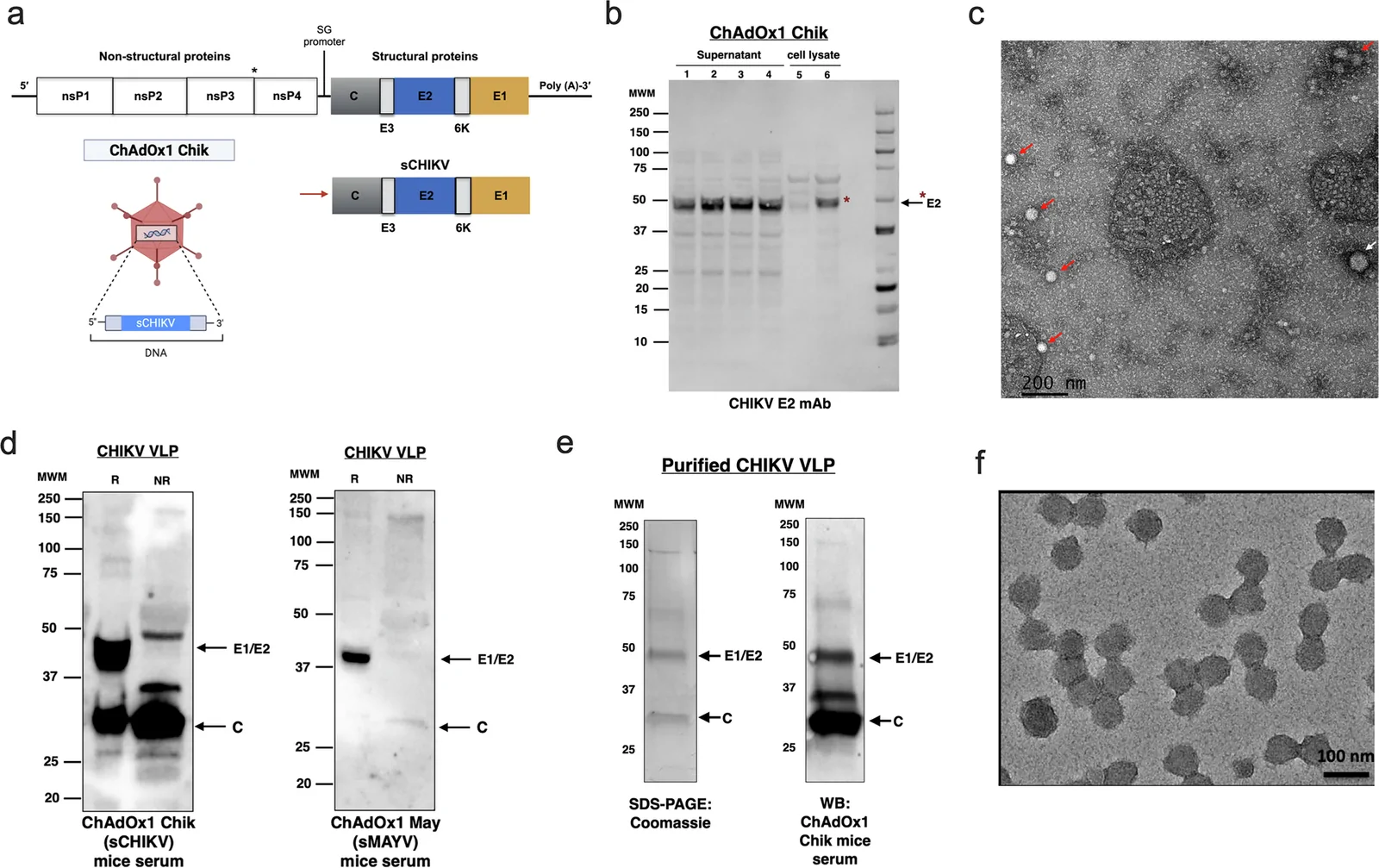
Production, purification and characterisation of CHIKV VLP. **a** A schematic representation of the CHIKV genome and the ChAdOx1 Chik vaccine construct encoding the structural polyprotein (sCHIKV: C, E3, E2, 6 K and E1). **b** Western blot analysis following infection of HEK293T cells with ChAdOx1 Chik. Cell lysates and supernatant were probed using anti-CHIKV E2 monoclonal antibody. **c** Transmission electron microscopy (TEM) image showing CHIKV VLPs in the supernatant of ChAdOx1 Chik infected HEK293T cells. Red arrows indicate CHIKV VLPs and white arrows indicate the larger ChAdOx1-Chik particles. **d** Western blot analysis of CHIKV VLP production in mammalian cells. Supernatants from HEK293T cells were concentrated using a 20% sucrose cushion centrifugation prior to WB analysis. WB were probed with sera from mice vaccinated with ChAdOx1 Chik (sCHIKV) under reduced (R) and non-reduced (NR) conditions. Sera from ChAdOx1 May (sMAYV) vaccinated mice were included here to demonstrate antigenic cross-reactivity between CHIKV and MAYV structural proteins rather than serving as a negative control. **e** SDS-PAGE (Coomassie staining) and WB of purified CHIKV VLP using sera from ChAdOx1 Chik vaccinated mice sera under reducing conditions. **f** Negative-stain TEM images of purified CHIKV VLPs.

To directly assess whether the ChAdOx1 Chik adenoviral vaccine platform produces CHIKV VLPs, we first infected HEK293T cells with ChAdOx1 Chik encoding the CHIKV structural polyprotein (sCHIKV: C, E3, E2, 6K, E1) (Fig. 1a). Expression of the CHIKV structural polyprotein was confirmed in both cell lysates and culture supernatants by western blot (WB) using an anti-CHIKV E2 monoclonal antibody, which detected a band around 45 kDa corresponding to the CHIKV E2 (Fig. 1b). Importantly, transmission electron microscopy (TEM) of supernatants from ChAdOx1 Chik-infected cells revealed VLPs with morphology consistent with CHIKV particles (Fig. 1c). These data demonstrate that the ChAdOx1 Chik vector itself can direct mammalian cells to produce CHIKV VLPs.

To complement these findings and further characterise VLP formation driven by the encoded structural polyprotein, sCHIKV was cloned into the pHLsec expression vector and transiently expressed in mammalian cells (Fig. 1d). Supernatant from HEK293T cells expressing the sCHIKV antigens was collected and concentrated through a 20% sucrose cushion. WB analysis using sera from ChAdOx1 Chik-immunised mice detected bands around 45 kDa and 33 kDa under reducing condition (R), corresponding to the expected sizes of CHIKV E1/E2 and capsid, respectively (Fig. 1d). Sera from ChAdOx1-May (sMAYV)-vaccinated mice was included to demonstrate partial antigenic cross-reactivity (rather than serve as a strict negative control) and this showed a similar band ~45 kDa (Fig. 1d).

For structural analysis, CHIKV VLPs were further purified by sucrose gradient centrifugation to obtain high-purity preparations. Purified VLPs were analysed by SDS-PAGE and WB (Fig. 1e) and negative-stain TEM confirmed the presence of uniform particles of approximately 700 Å (70 nm) in diameter (Fig. 1f). Together, these experiments demonstrate that both heterologous expression and the ChAdOx1 Chik vaccine vector can produce CHIKV VLPs in mammalian cells.

### CHIKV VLPs exhibit the same overall assembly as the infectious virion

**Fig. 2:**
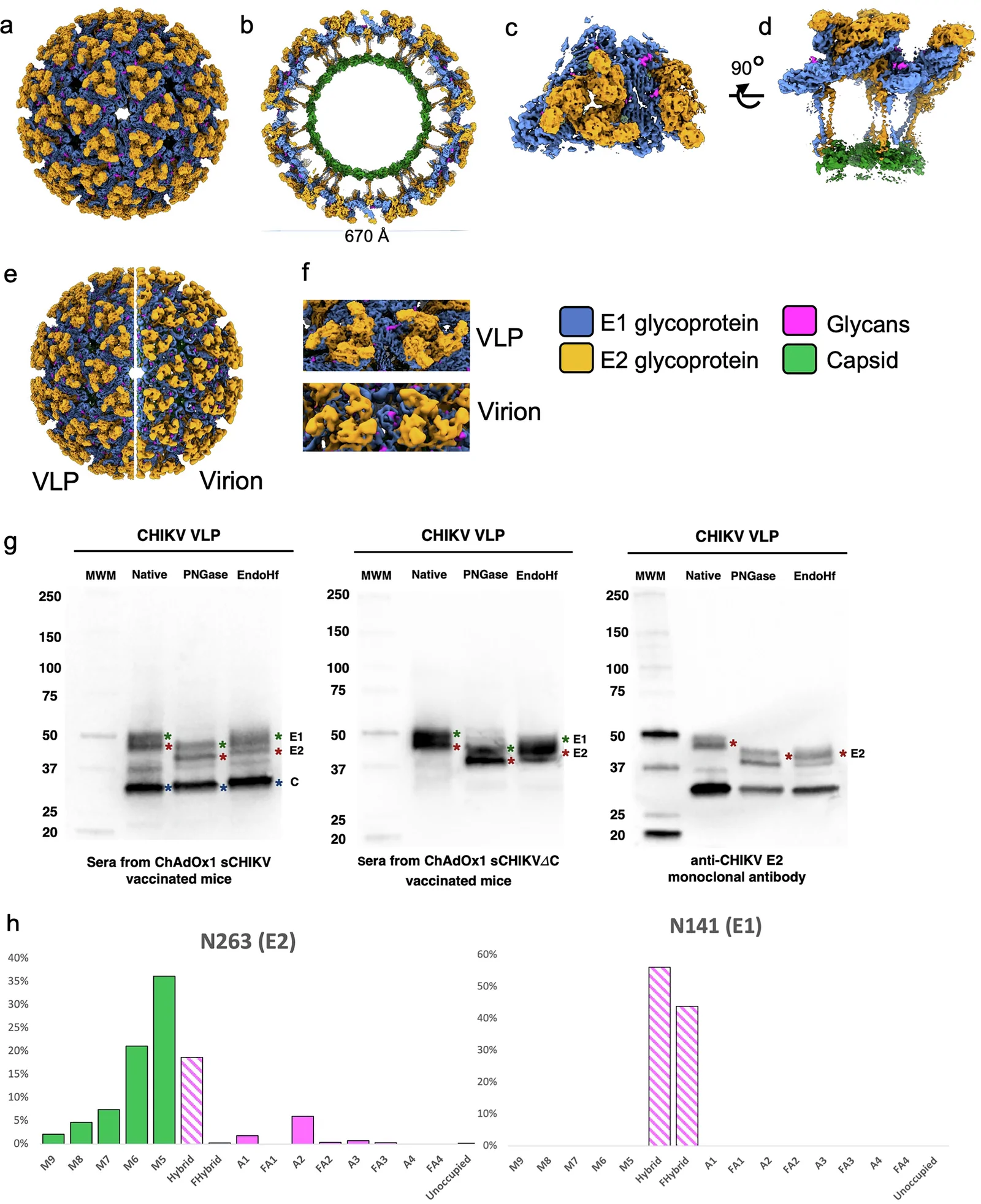
Cryo-EM structure and glycosylation analysis of CHIKV VLPs. **a** Structure of CHIKV-VLP visualised along the two-fold axis of icosahedral symmetry. **b** A central slice through the cryo-EM density map of the CHIKV VLP. **c** Close-up of one asymmetric unit composed of one full E1/E2 trimer around icosahedral five-fold symmetry axis and one third of a trimer at icosahedral three-fold axis. **d** The asymmetric unit shown in (**c**) rotated 90 degrees around the indicated axis. **e** CHIKV VLP (left) and the mature CHIKV virion (right, EMD-26446) are shown for comparison. **f** Close-ups views of the cryo-EM maps in (**e**) for the VLP and the virion. **g** N-linked glycosylation of purified CHIKV VLP produced in HEK293T cells assessed by western blot under reducing condition using sera from mice vaccinated with ChAdOx1 sCHIKV, ChAdOx1 sCHIKVΔC or anti-CHIKV E2 monoclonal antibody, before and after digestion with PNGase F or Endo-Hf. **h** N-glycan analysis by in-line liquid chromatography-mass spectrometry of glycopeptides. Green, pink (filled) and pink (lined) indicate oligomannose-type glycan, complex-type glycan and hybrid-type glycans, respectively. Complex glycans are grouped according to the number of antennae and the presence of core fucosylation (A1 to FA4).

Mature alphaviruses have a *T* = 4 icosahedral symmetry and consist of 80 envelope trimers of E1-E2 heterodimers (60 quasi-three-fold symmetry trimers and 20 icosahedral three-fold symmetry trimers) and 240 copies of capsid protein^39–41^. The ectodomain structure of the E2-E1 heterodimers of related alphaviruses Semliki Forest virus, Sindbis virus and CHIKV has been studied by X-ray crystallography^14,42–44^. In addition, cryo-EM was used to determine the complete structures of several alphaviruses^40,45–50^, including CHIKV virions and VLPs^13,43,51–55^. To assess whether our CHIKV VLPs exhibit the same architecture as wild-type virus, we determined the cryo-EM structure of our CHIKV VLP by single particle analysis (Fig. 2a). The localised reconstruction method was used to improve the resolution of the conventional icosahedral reconstruction of the E1–E2 trimers from 5.7 Å to 4 Å. Our CHIKV VLP structure was highly similar to the previously published CHIKV virion (EMD-26466)^55^, with a high cross-correlation coefficient of 0.92 (Fig. 2e), showing the characteristic *T* = 4 arrangement of the 80 glycoprotein trimers made of E1 and E2 glycoproteins in addition to the capsid protein layer (Fig. 2b-d). Additional densities corresponding to glycans are present in positions of E1 N141 and E2 N263 glycosylation sites similar to that of CHIKV virion structure reported earlier (Fig. 2c–f)^13,51–54^. In summary, these data confirms that our CHIKV VLP is structurally highly similar to structures of the native CHIKV virion determined at 8.2 Å^55^.

### CHIKV VLPs show authentic E1/E2 glycosylation

CHIKV glycoproteins, E1 and E2 are N-glycosylated and these N-glycans play a key role in virus pathogenesis by facilitating binding of CHIKV virions to host cell receptors^51,56–59^. To assess whether the antigenic surface presented by our CHIKV VLPs derived from ChAdOx1 Chik vaccine antigen resembles that of the native virion, we analysed the glycosylation status of purified CHIKV VLPs by LC-MS. The CHIKV E1 and E2 proteins each were predicted with high residue score to encode an N-linked glycosylation sequon (NXT/S, where X ≠ P) at N141 and N263, respectively by GlycoEP^60^. In addition, E2 encodes N-linked sequons at N273 and N345, but both N273 and N345 had a low probability of occupancy^60^.

To investigate N-glycosylation experimentally, a series of Western blot analyses was performed on purified CHIKV VLP, before and after de-glycosylation, using sera from mice vaccinated with ChAdOx1 sCHIKV, ChAdOx1 sCHIKVΔC (sCHIKV lacking capsid) as well as an anti-CHIKV E2 monoclonal antibody (Fig. 2g). WB of native CHIKV VLP using sera from ChAdOx1 sCHIKV vaccinated mice showed bands at ~47.5 kDa, 45.2 kDa and 29.6 kDa, corresponding to the predicted molecular masses of E1, E2 and capsid, respectively whereas sera from ChAdOx1 sCHIKVΔC vaccinated mice detected only E1 and E2 bands. PNGase F digestion resulted in the expected reduction in the molecular mass of CHIKV E1 and E2, consistent with N141 and N263 being N-glycosylated. Moreover, a reduction in the molecular mass of E2 was also observed following Endo-Hf digestion (albeit less marked than with PNGase F), suggesting the presence of high-mannose-type glycosylation at this site.

Consistent with these results, analysis of glycan composition by in-line LC-MS confirmed that the N141 site on the E1 glycoprotein (N950 on structural polyprotein; sCHIKV) was occupied exclusively by hybrid-type glycosylation, whereas the N263 site on the E2 glycoprotein (N588 on sCHIKV) carried a mixture of predominantly high mannose glycans, with a minor population of hybrid-type and complex-type glycans (Fig. 2h). This analysis indicated nearly full occupancy of both N-linked glycosylation sites. In summary, these data show that the glycosylation of our CHIKV VLP antigen derived from the ChAdOx1 Chik vaccine is representative of typical alphavirus glycoprotein glycosylation.

### Unadjuvanted VLPs significantly reduced viremia and CHIKV-induced foot swelling in mice

**Fig. 3:**
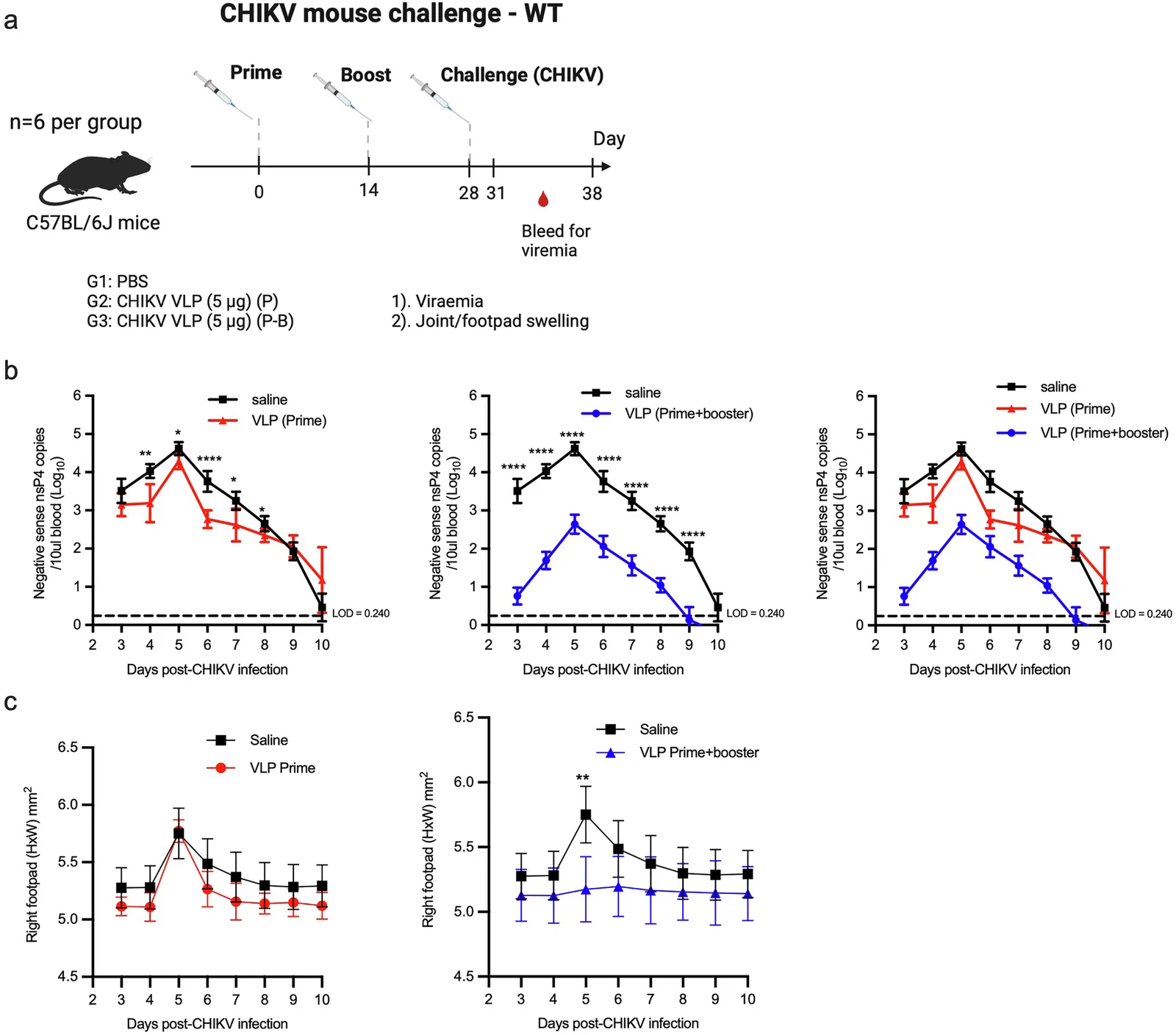
Assessment of protective efficacy induced by CHIKV VLP vaccines. **a** Non-adjuvanted CHIKV VLP (either prime only or prime boost) vaccinated C57BL/6 mice were inoculated subcutaneously in the right hind footpad with 5 × 10^6^ PFU CHIKV (LR2006 strain) at 4 weeks post-immunisation. After CHIKV challenge, viraemia was monitored daily from 3-day post-infection (dpi) until 10 dpi. Joint swelling of the footpad was scored starting at day 0 and 3 to 10 dpi. **b** viral load kinetics of CHIKV challenge groups were monitored to assess the protective efficacy. Graphs show days post-challenge on the x-axis versus viral loads on the y-axis. **c** Right footpad swelling of CHIKV challenge groups were monitored to assess protective efficacy. Data represented as mean, and SD. *P*-values were determined by using unpaired T-test, *p < 0.05, **p < 0.01, ****p < 0.0001.

The FDA-approved VIMKUNYA vaccine contains HEK293 cell-derived VLPs (CHIKV Senegal strain 37997) adsorbed on aluminium hydroxide as adjuvant^24^. However, previous studies with mammalian and baculovirus system-derived CHIKV VLP demonstrated good protective efficacy in pre-clinical challenge studies in mice and NHPs without use of adjuvant^61,62^. To confirm the protective efficacy of our purified CHIKV VLP as an unadjuvanted vaccine candidate, C57BL/6 mice (*n* = 6) were immunised intramuscularly with one or two doses of unadjuvanted 5 µg of CHIKV VLPs and subcutaneously challenged with 5 × 10^6^ PFU CHIKV (LR2006 strain) four-weeks post-immunisation (Fig. 3a). Viremia and swelling at the inoculation sites were monitored as these are key hallmarks of arthritogenic alphavirus infection.

In PBS-vaccinated control mice, mean peak viremia reached 4.62 log_10_ copies/10 µl blood on day 3–5 days post-CHIKV infection (Fig. 3b). In contrast, mean peak viremia on day 3-5 in the CHIKV VLP (prime) and CHIKV VLP (prime-boost) groups was 4.28 and 2.63 log_10_ copies/10 µl, respectively. This corresponds to approximately 0.4 log_10_ and 2.0 log_10_ reduction in peak viremia compared with PBS controls. Thus, vaccination with either one or two doses of unadjuvanted 5 µg CHIKV VLP provided statistically significant protection against viremia. In addition, two doses of CHIKV VLPs conferred protection against CHIKV-induced foot swelling. On day 5 post-challenge, when swelling peaked in PBS controls, the mean footpad area in PBS-vaccinated mice was 5.75 mm^2^, compared with 5.18 mm^2^ in the prime-boost CHIKV VLP group, corresponding to a statistically significant reduction of 0.57 mm^2^ (Fig. 3c). Although, a single dose of VLPs did not result in a statistically significant difference in foot swelling on day 5, swelling in the prime group remained consistently lower than in PBS controls across days 2–10 post-infection (Fig. 3c).

### Comparative immunogenicity evaluation of vaccine platforms reveals all can induce high CHIKV specific antibody titres without use of adjuvant

We next sought to compare the immunogenicity of three CHIKV vaccine platforms based on the same structural antigen: VLPs, viral vectors and recombinant protein. We have previously developed ChAdOx1 Chik and MVA-Chik viral-vectored vaccines and shown that ChAdOx1 Chik provides full protection against CHIKV challenge in A129 mice with induction of highly neutralising antibody titres while MVA-Chik served as an effective booster following prime vaccination with ChAdOx1 Chik^34,36^. Heterologous adenoviral-MVA prime-boost regimens are known to induce strong and long-lasting multifunctional T cell responses in other infectious disease settings such as malaria or Ebola^63–65^, but this has not been directly compared with VLP or protein-based CHIKV vaccines in a head-to-head manner.

**Fig. 4:**
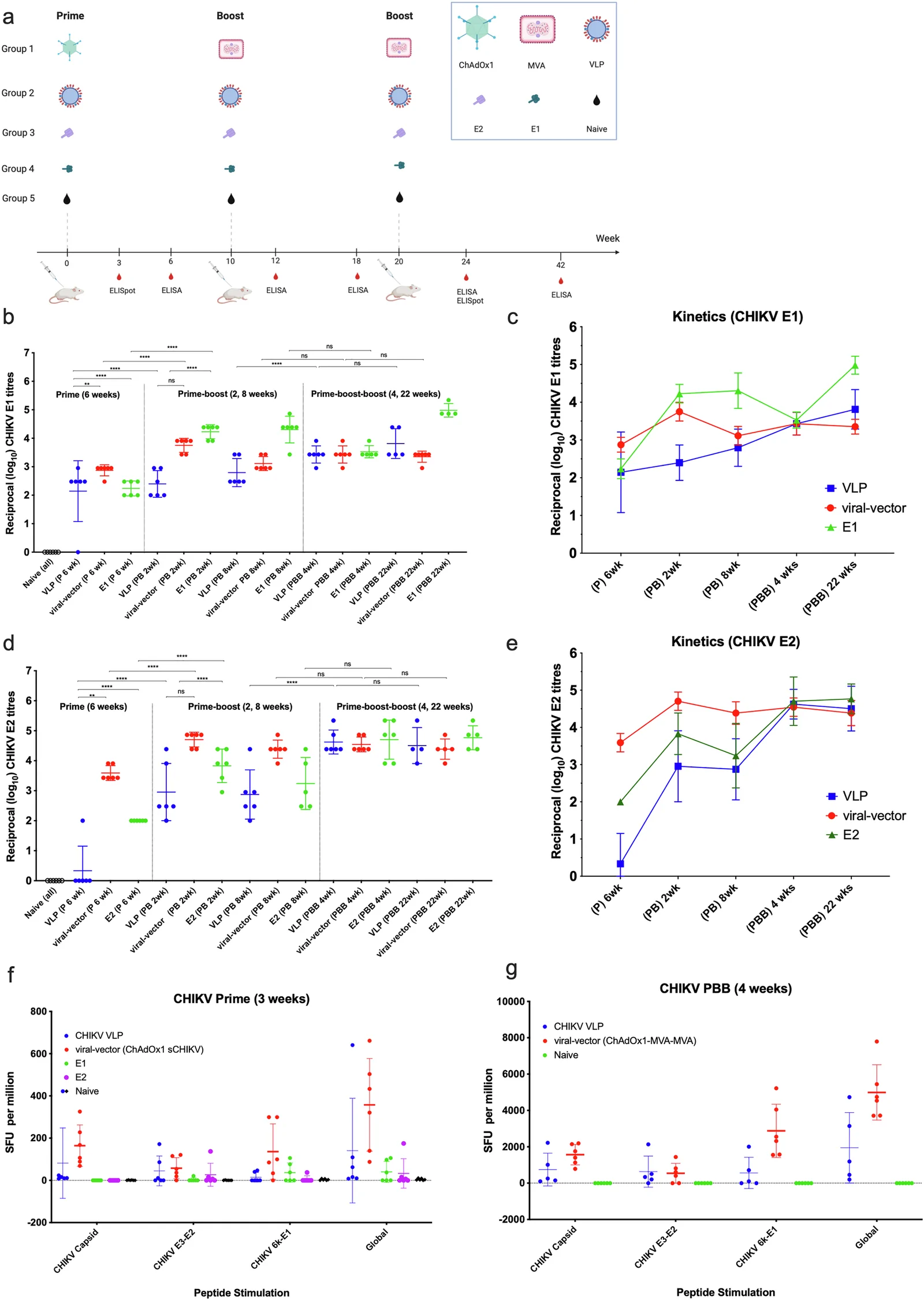
Comparative assessment of CHIKV vaccine platforms in BABL/c strain of mice. **a** BALB/c mice (*n* = 6) were immunised intramuscularly (IM) according to the prime-boost-boost schedule shown, and blood sampling time points for ELISA and ELISpot assays are indicated. Vaccine groups and their respective priming and boosting agents are shown. Humoral immune responses elicited by CHIKV VLP, viral-vectored vaccines and E1 or E2 sub-unit vaccine. CHIKV E1 or E2 ELISAs were performed on sera collected at 6 weeks after the prime immunisation, 2 weeks and 8 weeks after P-B and 4 and 22 weeks after P-B-B (**b** and **d**) and the kinetics of anti-CHIKV E1 or E2 titres over time are shown in (**c**) and (**e**). Group comparisons in (**b**) and (**d**) were analysed using one-way ANOVA with Tukey’s multiple comparisons test. Cellular immune responses elicited by CHIKV VLP, viral-vectored vaccines and E1 or E2 sub-unit vaccines. PBMCs were stimulated with a peptide pool spanning the CHIKV structural polyprotein and IFNγ producing cells were quantified by ELISpot at (**f**) 3 weeks post-prime and (**g**) 4 weeks post-prime-boost-boost (P-B-B). Values represent spot-forming cells (SFU) per million PBMCs. Overlapping 20-mer peptides spanning the CHIKV structural polyprotein (10 µg/mL) were used for stimulation. Statistical analysis was performed using one-way ANOVA with Tukey’s multiple comparisons test. Line colours represent individual vaccine groups; mean antibody titres or SFUs +/- error bars are shown by solid coloured lines.

To evaluate and compare the immunogenicity of these platforms and to investigate their ability to induce strong and long-lasting immune responses, BALB/c mice (*n* = 6) were immunised intramuscularly with CHIKV VLP, viral-vectored sCHIKV (ChAdOx1-MVA-MVA Chik), soluble CHIKV E2 and E1 antigens in a non-adjuvanted prime-boost-boost (P-B-B) regimen (Fig. 4). This design allowed us to assess how the same CHIKV structural antigen performs when delivered as a viral vector, VLP or soluble protein and to determine whether VLPs and viral vectors can match or surpass soluble E2 in inducing CHIKV-specific and neutralising antibody responses. The immunogenicity of these CHIKV vaccine candidates was assessed by monitoring of anti-CHIKV E2 and anti-CHIKV E1 titres by ELISA at multiple time points following prime (P), prime-boost (P-B) and prime-boost-boost (P-B-B) immunisations. As the immunogenicity of protein-based vaccines (VLP and E1 or E2) is predominantly humoral, ELISpot assays were performed at two time points only (3 weeks post-prime and 4 weeks post P-B-B) to confirm T-cell responses.

ELISA assays were performed by coating 96-well plates with CHIKV E1 or CHIKV E2 antigens to monitor the levels of anti-CHIKV E1 and E2 in mice after immunisations respectively (Fig. 4b-e). At 6 weeks post-prime immunisation, the anti-CHIKV E1 titres were detected in all vaccine candidates; mean titre values of VLP, ChAdOx1 sCHIKV and E1 sub-units were 2.14, 2.87 and 2.24, respectively. At 2 weeks following prime-boost (P-B) immunisation, the mean anti-E1 titres of VLP, ChAdOx1 sCHIKV and E1 sub-units increased to 2.40, 3.75 and 4.23 followed by a slight increase in mean antibody titres for VLP (mean titres = 2.80) and E1 (mean titres = 4.3) at 8 weeks PB while there was a slight decline in antibody titres in ChAdOx1 sCHIKV to mean titres of 3.11 (Fig. 4b). The mean antibody titres of VLP and ChAdOx1 sCHIKV increased further to 3.43 at 4 weeks after prime-boost-boost (P-B-B) immunisation (Fig. 4c). Interestingly, the levels of anti-CHIKV E1 titres for E1 group (mean titres of 3.53) did not increase further up on the second boost at 4 weeks P-B-B. The anti-E1 titres remained high (3.81, 3.35, 4.98) in all groups even after 22 weeks of PBB immunisation. The kinetics of anti-E1 titres over time are shown in Fig. 4c, which highlights that the mean antibody titres for VLP continued to increase up on boosting while the first MVA boosting effect may be greater than the subsequent boosts with MVA.

The anti-CHIKV E2 titres were measured in the same manner which showed that ChAdOx1 sCHIKV was the best at inducing anti-E2 antibodies post prime immunisation with mean titre values at 3.59 post-prime 6 weeks (Fig. 4d, e). The mean titres for VLP and E2 were 0.33 and 2.00 after 6 weeks post-prime immunisation. However, upon the boost, the mean antibody titres for all groups (VLP, ChAdOx1 sCHIKV and E2 sub-units) increased significantly to 2.95, 4.70 and 3.83, respectively. The mean antibody titres for all groups maintained at high levels 8 weeks after P-B with no statistical difference in the mean titres between the groups (Fig. 4e). These mean antibody titres were further increased by second boost which maintained at high levels >4.39 for all groups at 22 weeks after P-B-B immunisation (Fig. 4e). These results suggest that the viral-vectored platform is best at inducing the anti-E2 responses early which may be important in protective efficacy against CHIKV infections, but VLP and sub-unit vaccines reach similar antibody titres upon boost immunisation.

As previous studies have demonstrated the protective role of cellular immunity against CHIKV^66–68^, cellular responses elicited by CHIKV vaccine candidates after 3 weeks post-immunisation and 4 weeks post-prime-boost-boost (P-B-B) immunisations were assessed by Interferon-γ (IFNγ) enzyme-linked immunospot (ELISpot) assay using PBMCs (Fig. 4f, g). At 3 weeks post-prime, BALB/c mice receiving ChAdOx1 sCHIKVand CHIKV VLP induced modest IFNγ-producing global T cell responses (mean values of 358 and 141 SFU/10^6^ PBMCs respectively), while the protein based sub-unit vaccine candidates (E1 and E2) induced minimal amount of IFNγ-producing T cell responses (mean values close to 30 SFU/10^6^ PBMCs) as expected (Fig. 4f). At 4 weeks post P-B-B immunisation, the viral-vectored group showed high T-cell responses after stimulation with the sCHIKV peptide pool (mean global T-cell responses = 4991 SFU/10^6^ PBMCs) while CHIKV VLP group showed good level of T-cell response (mean global T- cell responses = 1945 SFU/10^6^ PBMCs) (Fig. 4g). The majority of T-cell responses were directed towards the CHIKV 6K-E1 peptide pool (mean=2878 SFU/10^6^ PBMCs) and CHIKV capsid peptide pool (mean= 1569 SFU/10^6^ PBMCs) with smaller responses contributed by CHIKV capsid region (mean =545 SFU/10^6^ PBMCs) in mice vaccinated with viral-vectored vaccines. For the CHIKV VLP-vaccinated group, the mean T-cell responses towards capsid, E2-E2 and 6k-E1 were more similar at 746, 636 and 563, respectively.

### Comparative evaluation of virus neutralisation reveals only the viral-vectored and VLP vaccines can induce neutralising antibodies without adjuvant

**Fig. 5:**
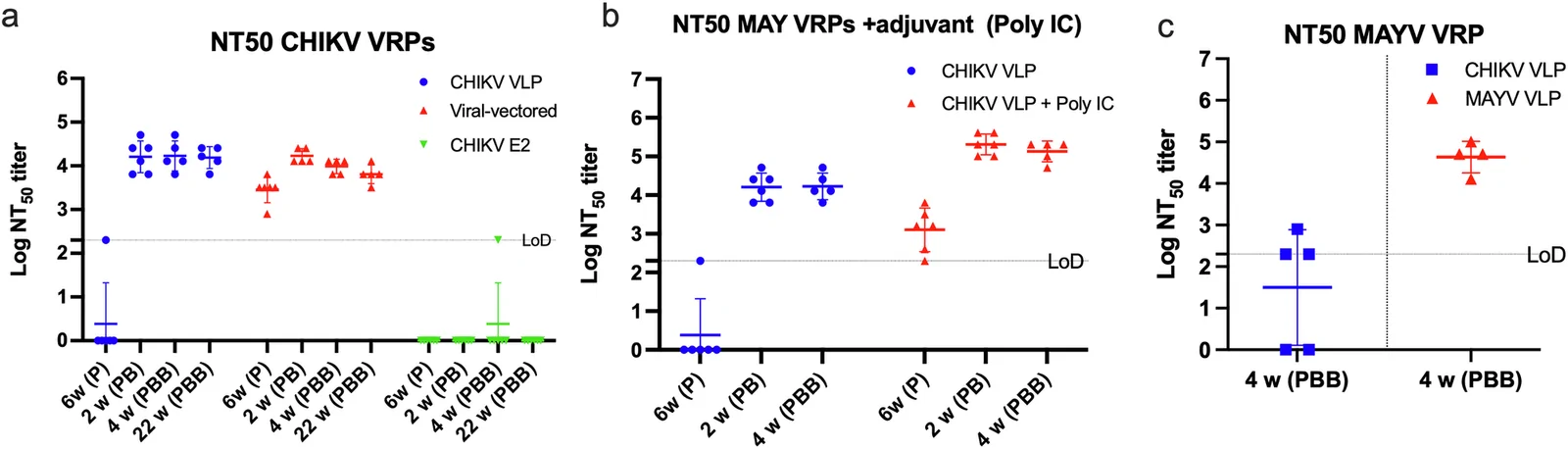
Neutralisation potency of CHIKV vaccine platforms. **a**–**c** CHIKV and MAYV VRP neutralisation assays. CHIKV NT_50_ titres for BALB/c mice serum samples post immunisations with CHIKV VLP, viral-vectored sCHIKV or E2 are shown. MAYV VRP neutralisation assay was used to assess cross-neutralisation antibody responses induced by CHIKV VLPs. In (**c**), MAYV VLP NT50 titres (red triangle) obtained using anti-MAYV VLP sera (generated in our previous study^76^, are shown as positive control; these assays were performed at the same time and are included here for reference and comparison at the antibody levels induced by CHIKV VLP. Coloured lines represent the mean with SD and error bars. *P* values in (**b**, **c**) were determined by one-way ANOVA and Tukey’s multiple comparisons test. *p* > 0.05 (ns), *p* < 0.01 (**), *p* < 0.0001 (****). *P* value in (**c**) was determined by the Mann-Whitney *U* test. *p* > 0.05 (ns).

In this study, we have used well-established CHIKV neutralisation assay based on CHIKV virus replicon particles (VRPs) to assess neutralising capacity of our CHIKV vaccine candidates (VLP, viral-vectors and E2 antigen)^34,69^. We determined the NT50 values for BALB/c mice sera obtained after vaccination with CHIKV VLP, viral-vectors and E2 subunits at prime (6 weeks), prime-boost (2 weeks) and prime-boost-boost (4 weeks and 22 weeks) timepoints. At 6 weeks post-prime immunisation, sera from ChAdOx1 sCHIKV vaccinated mice demonstrated high neutralisation titres with reciprocal log NT50 titres of 3.45 (Fig. 5a). This was significantly higher than mice vaccinated with CHIKV VLP (mean NT50 = 0.39) where only 1 out of 6 mice had detectable NT50 titres and mice vaccinated with E2 had no detectable NT50 titres. Following a boost, the mean NT50 titres for CHIKV VLP and viral-vectored groups increased significantly to 4.21 and 4.23, respectively. CHIKV E2 groups did not have any detectable NT50 titre in any mice. After 4 weeks prime-boost-boost immunisations, the mean NT50 titres for VLP and viral-vectored (ChAdOx1-MVA-MVA) groups did not increase any further. Notably, one out of six mice in CHIKV E2 group had a detectable NT50 titres following the second boost with CHIKV E2. To assess the durability of neutralising antibodies, mice sera after 22 weeks post-PBB vaccination were tested which demonstrated the mean NT50 titres of both CHIKV VLP and viral vectors remained high at 4.19 and 3.80, respectively.

Next, we tested if higher neutralising antibodies can be induced by adjuvanted CHIKV VLP. Poly(I:C) was used in this study as it mimics viral infections and shown to be a potent adjuvant to boost immune responses in vaccines against viral diseases such as influenza, SARS-COV and HIV by activating Toll-like Receptor 3 (TLR3)^70–74^. We determined the NT50 values for BALB/c mice sera obtained after vaccination with CHIKV VLP + Poly (I:C) adjuvants at the same timepoints (prime 6 weeks, prime-boost 2 weeks and prime-boost-boost 4 weeks) to allow the comparison to the NT50 titres determined by vaccinating CHIKV VLP on its own as was shown in Fig. 5b. Interestingly, the NT50 titres for CHIKV VLP + Poly (I:C) adjuvanted group was higher at all timepoints. All the mice vaccinated with CHIKV VLP + Poly (I:C) adjuvant showed detectable mean NT50 titres (mean NT50 titres of 3.10) post-prime immunisation and the mean NT50 titres increased significantly to 5.11 upon first boosting with VLP. The second boost with CHIKV VLP + Poly IC did not increase the mean NT titres further (mean NT50 titres 5.13) at the 4 weeks post-PBB. Taken together, our data suggest that CHIKV NT50 titres induced by CHIKV VLP can be increased with use of Poly (I:C) adjuvant with its effect most prominent particularly post-prime immunisation (after a single dose of vaccination).

Next, to assess the ability of CHIKV VLPs to provide cross-neutralisation against MAYV, a related arthritogenic alphavirus, we performed a MAYV VRP neutralisation assay using mice sera obtained after 4 weeks post-PBB vaccination of CHIKV VLP^75^. As the CHIKV NT50 titres at 4 weeks-PBB were both high for CHIKV VLPs administered with or without Poly (I:C) (4.23 and 5.13, respectively), we used sera from the non-adjuvanted CHIKV VLP group in this MAYV VRP neutralisation assay. Interestingly, despite high NT50 against CHIKV, only 3 out of 5 mice had detectable NT50 titres against MAYV (mean NT50 titres of 1.51), in contrast to the high NT50 titres against MAYV induced by MAYV VLP-vaccinated mice from our previous study (positive control; included in Fig. 5c for reference and comparison). These results suggest that despite robust specific CHIKV-specific neutralisation induced by CHIKV VLPs, cross-neutralising antibody responses against MAYV are limited. This was unexpected, as our previous study showed that mice sera obtained after 4 weeks PBB vaccination of MAYV VLP and viral-vectored MAYV vaccines exhibited high cross-neutralisation against CHIKV (mean NT50 titres of 3.05 and 3.51, respectively)^76^. Taken together, our results confirm that our CHIKV VLP and viral-vectored vaccines can elicit functional neutralising antibodies against CHIKV. Our data also suggests that neutralisation capacity of CHIKV VLP can be significantly increased by use of adjuvant particularly when CHIKV VLP vaccine is given as a single dose but additional adjuvanted boosts conferred no further benefits, indicating use of adjuvants may not be needed in multi-dose CHIKV VLP vaccination regimens.

## Discussion

To confirm our hypothesis that our clinically relevant ChAdOx1 Chik will direct host cells to secrete the encoded structural polyprotein in the form of VLPs that closely mimic authentic CHIKV virions, thereby presenting both linear and conformation epitopes required for potent antibody-mediated protection, we produced CHIKV virus-like particles (VLPs) in mammalian cells using the same codon-optimised sequence of CHIKV structural polyprotein in our ChAdOx1 Chik vaccine. Production of CHIKV VLP has been reported in multiple expression systems including mammalian, insect and yeast cells^61,77,78^. In each of these expression systems, they were shown to be highly immunogenic and provided protection against CHIKV infection in different animal models of CHIKV-induced disease^61,77,78^. To demonstrate that our CHIKV VLPs closely resemble the native virion, the structure of CHIKV VLPs was determined by Cryo-EM single particle three-dimensional reconstruction in this study. The CHIKV VLP structure was found to be highly similar to the CHIKV virion structure published earlier showing the characteristic *T* = 4 arrangement of the 80 glycoprotein trimers made of E1 and E2 glycoproteins in addition to the capsid protein layer^13,51–55^. Analysis of N-linked glycosylation of CHIKV VLP revealed that sites E947 and N271, from the E1 and E3 glycoproteins, respectively, consisted of complex-type glycans, while E2 glycoprotein site N586 had mixture of oligomannose, hybrid and complex-type glycans. This glycan composition seems to largely match that observed for other alphaviruses, such as SFV and EEEV where E2 glycoprotein contained conserved under-processed oligomannose-type glycans while E1 contained predominantly highly processed glycans such as complex-type and paucimannose-type glycosylations suggesting that the glycosylation of our CHIKV VLPs is representative of the native virus^57,79,80^. Combined with the high level of structural resemblance of the envelope glycoproteins, these combined data indicate that our CHIKV VLPs present an antigenic surface closely resembling that of the native virus.

To confirm the protective efficacy of our purified CHIKV VLP as a vaccine candidate without an adjuvant, one or two doses of unadjuvanted CHIKV VLP were evaluated in mice challenge model. Our data suggests that vaccination with either single or two doses of non-adjuvanted 5 µg of CHIKV VLP can provide statistically significant protection against viremia. In addition, two doses of CHIKV VLPs were able to provide protection against CHIKV-induced foot swelling suggesting that CHIKV VLP could provide protective efficacy without an adjuvant. Our data is in agreement with previous studies with mammalian and baculovirus system derived CHIKV VLP which demonstrated good protective efficacy in pre-clinical challenge studies in mice and NHPs without use of adjuvant^61,62^.

As few head-to-head comparisons of adjuvant-free CHIKV vaccine platforms have been reported, we performed a comparative evaluation of CHIKV platforms expressing the equivalent antigens in either viral-vectored, or VLP or sub-unit vaccines in mice. The humoral immunogenicity results indicate that the levels of anti-E1 and anti-E2 antibodies elicited by CHIKV VLPs post-prime immunisation were lower than viral vectored vaccine (ChAdOx1 sCHIKV). This may be due to lower doses of VLPs used for initial prime vaccination (mice were primed with 2 µg and boosted with 5 µg of VLP) and the absence of an adjuvant. By contrast, previous pre-clinical CHIKV VLP studies in mice commonly used higher VLP doses (10–40 µg) formulated with Freund’s adjuvant to induce robust neutralising antibody responses^78^. It would be therefore be of interest in future work to test whether higher doses of CHIKV VLPs and inclusion of aluminium hydroxide, as used in the licensed VIMKUNYA vaccine, could further increase immunogenicity and allow more direct comparison with ChAdOx1 Chik after a single prime. T cell immunogenicity elicited by ChAdOx1/MVA regimen was significantly higher than that induced by CHIKV VLPs. However, following prime-boost or prime-boost-boost immunisations, anti-E1 and E2 antibody titres in the CHIKV VLP group were comparable to those induced by the viral vectored vaccines and sub-unit vaccines. CHIKV neutralisation was used to further assess the neutralising capacity of CHIKV VLP, viral-vectors and E2 subunit vaccines. CHIKV VLPs induced high neutralisation titres comparable to ChAdOx1-sCHIKV after two doses of non-adjuvanted immunisation and these neutralising titres remained high up to 22 weeks post-PBB immunisation. In contrast, CHIKV E2 subunit vaccines failed to induce detectable neutralising antibody titres.

We have previously demonstrated that the neutralisation titres induced by MAYV VLP with or without Poly (I:C) adjuvant were similar, suggesting that MAYV VLPs can induce high neutralisation titres without use of an adjuvant^76^. In the present study, however all mice vaccinated with CHIKV VLPs + Poly (I:C) showed a significant increase in mean NT50 titres (mean NT50 titres of 3.10) after a single prime, which further increased to 5.11 after the first boost. A second boost with CHIKV VLP + Poly (I:C) did not increase the mean NT titres further (mean NT50 titres 5.13 at the 4 weeks post-PBB). Taken together, our data suggest that CHIKV NT50 titres induced by CHIKV VLP are significantly enhanced by Poly (I:C) adjuvant with the most pronounced effect observed after the prime dose.

We evaluated the cross-neutralising ability of mice sera obtained after 4 weeks post-PBB vaccination of CHIKV VLP and surprisingly, despite high neutralisation against CHIKV, only 3 out of 5 mice had detectable NT50 titres against MAYV which suggest that there may be limited cross-neutralising antibodies against MAYV. Notably, in a previous study, we also assessed the cross-protective potential of ChAdOx1 Chik against MAYV challenge in mice, where a single vaccine dose conferred partial cross-protection: three out of five vaccinated animals survived MAYV infection, although foot inflammation was not reduced or delayed despite some reduction in viremia^35^. The reason for the lack of neutralising antibodies in the CHIKV E2 sub-unit vaccine could be due to absence of adjuvant to boost the immune responses or the absence of conformational epitopes, which may be essential in providing efficacy. Previous studies comparing the immunogenicity and protective efficacy of the CHIKV E1 and E2 subunits, and the CHIKV VLP in AG129 mice, have shown that upon prime-boost immunisations of CHIKV VLP subcutaneously with Maxtrix M adjuvant induced high neutralising antibody titres^62^. In addition, CHIKV VLP provided complete protection against lethal CHIKV challenge in mice whereas CHIKV E1 and E2 resulted in lower levels of neutralising antibodies with partial protection^62^.

In this work, our clinically relevant ChAdOx1 Chik vaccine was shown to self-assemble into VLPs in mammalian cells which combines the advantages of leading VLP platforms (induction of conformational epitopes) with the intrinsic immunostimulatory and T-cell priming properties of adenoviral vectors providing the mechanism of its vaccine-induced protection demonstrated in pre-clinical and clinical trials^34–37^. Its ability to induce robust neutralising antibody responses without the use of aluminium adjuvant may help address public concerns around cumulative aluminium exposure in childhood immunisation schedules and offers an alternative vaccine platform for protection against CHIKV. Our data demonstrated that Poly (I:C) adjuvant enhanced neutralising titres after a single VLP dose, but additional adjuvanted boosts conferred no further benefits, indicating use of adjuvants may not be needed in multi-dose CHIKV VLP vaccination regimens. This support the vaccination with CHIKV VLP + adjuvant particularly for a single dose of vaccine which is in line with licensed VIMKUNYA which contains 40 µg of CHIKV VLP with aluminium hydroxide and is currently licensed for individuals 12 years and older. Given some public concerns such as neurotoxicity, MMF and late-onset persistent asthma associated with chronic exposure to aluminium by vaccination, it may be interesting to test CHIKV VLP vaccine without an adjuvant can induce comparable immunogenicity in adults and children (< 12 years old) upon booster dose (prime-boost regimen) as shown in this study which may support multi-dose CHIKV VLP regimen without an adjuvant.

## Methods

### Design and cloning of CHIKV structural transgene

The full-length structural polyprotein sequence of CHIKV(C, E3, E2, 6K, E1) was synthetically generated by GeneArt following codon optimisation for efficient expression in mammalian cells and inclusion of a Kozak sequence to improve the initiation of translation as previously described^35^. To clone the transgenes into the pHLsec expression plasmid without its secretory signal, an EcoRI site was included in the forward primer and an Acc65I restriction site was inserted into the reverse primer with a stop codon to prevent expression of His-tag in the pHLsec plasmid^81^. The plasmids containing sCHIKV fragment with new restrictions sites were PCR-cloned using AccuPrime^TM^ Pfx SuperMix (Invitrogen^TM^) as recommended by the manufacturer and subsequently ligated into pHLsec expression plasmid. The recombinant DNA plasmids were purified from *E. coli* using the miniprep kit (Qiagen, Hilden, Germany) and the content of each plasmid was verified by restriction analysis and DNA sequencing (Source, BioScience).

Forward primer (CTGAAGAATTCGCCACCATGGATTTTCTGCC)

reverse primer (GCTTGGGTACCTCATCATCTTCTCAGGGTGATACAG).

### Viral vector production, small-scale expression of ChAdOx1 Chik and characterisation by WB and TEM

Viral-vectors (ChAdOx1, and MVA) encoding the CHIKV structural cassette sCHIKV (C,E3,E2,6 K,E1) was produced as previously described^35,75^. Expression screening was performed in HEK293T cells by transduction with ChAdOx1 Chik at an MOI = 10. To analyse both secreted and intracellular antigens, supernatants and cell lysates were collected at 24 h and 48 h post-transduction. Samples were analysed by Western blot using a commercial anti-CHIKV E2 monoclonal antibody (Medix Biochemica, HM586; 1:1000) following the protocol previously described^75^ and outlined in ‘Expression and purification of CHIKV VLP from mammalian cells’ to detect expression of CHIKV structural polyproteins. To visualise the CHIKV VLPs produced following ChAdOx1 Chik transduction, transmission electron microscopy (TEM) was performed. Freshly glow-discharged TEM grids (C267, TAAB) were placed carbon-side down onto 10 µl droplets of sample and incubated for 2 min. The grids were then blotted and incubated for 10 seconds on a 20 µl droplet of aqueous uranyl acetate 2% (w/v), then blotted and allowed to air dry. Grids were imaged using a JEOL JEM-2100Plus 200 kV TEM equipped with a Gatan Oneview camera.

### Expression and purification of CHIKV VLP from mammalian cells

The large-scale transfection of CHIKV VLP was carried out in roller bottles using the standard PEI transfection of HEK293T cells as previously described^82^. Briefly, HEK293T cells were grown in DMEM (Sigma Aldrich, UK) supplemented with 10% FCS, 1 mM glutamine and 1x non-essential amino acids in roller bottles (Greiner Bio One, UK) at 37 °C for 3–4 days at 30 rpm until the cells reach ~ 90% confluency. The standard transfection was performed by incubating 0.5 mg of plasmid DNA with 1 ml of 1 mg/ml polyethylene-imine (PEI) for 10 min in a final volume of 50 ml serum free growth media per each roller bottle. The mixture was then added to the cells that contained 200 ml of fresh growth media and incubated at 37 °C with the bottle rotating at 30 rpm for 4-5 days. The supernatant was harvested by centrifuging at 3000 × *g* for 20 min to remove cell debris, and filtering through a 0.22-μm Stericup Filter (Millipore, UK). The expression of CHIKV VLP was tested by Western blot with mice sera vaccinated with ChAdOx1 Chik (sCHIKV) vaccine.

To further purify VLPs for structural studies, secreted protein fractions were precipitated by using PEG virus precipitation kit (Abcam, ab 102538) according to the protocol from the manufacturer and loaded on a sucrose gradient 20-60% prepared by gradient master (BioComp Instruments). Sucrose gradients were centrifuged at 28,000 rpm (SW40 Ti rotor, Beckman) for 16 h at 4 °C. The white band containing VLPs was isolated and resuspended in 10 ml STE buffer. The VLPs were pelleted by centrifugation through 20% sucrose cushion with 30000 rpm for 2 h at 4 °C. The pellet was resuspended in STE buffer and was assessed for the presence of CHIKV VLPs by SDS-PAGE and WB analysis (section 4.4). The integrity of VLPs was checked by TEM (section 4.5). The VLPs were stored at –80°C for future use.

### Characterisation of VLP by SDS-PAGE and WB

The protein composition of purified CHIKV VLPs was analysed by SDS-PAGE and Western blot (WB) using 1:500 dilutions of mice sera following a vaccination with ChAdOx1 Chik (sCHIKV) using previous described protocol^75^. Briefly, purified VLPs were added to Laemmli sample buffer (Bio-Rad) containing 20 mM β-mercaptoethanol. Samples were boiled for 4 min and loaded on a Mini-PROTEAN TGX protein gel (reducing conditions) with a protein marker (Bio-Rad, Precision Plus Protein^TM^ WesternC^TM^ standards) in 1x Tris-glycine-SDS running buffer (Bio-Rad). For samples under non-reducing conditions, non-reducing sample buffer was used instead. All samples were run at 200 V for 30-40 min. For Coomassie Blue stain, the gel was stained with Instant Blue Coomassie stain for 1 h and de-stained in dH_2_O before imaging. For Western blot (WB), proteins were transferred onto nitrocellulose membranes (Bio-Rad Trans-Blot® TurboT^M^). Membranes were blocked with 5% skim milk in 0.1% PBS/T for 1 h and then incubated with 1:500 dilutions of mice sera following a vaccination with ChAdOx1 Chik (sCHIKV). Sera from ChAdOx1 May (sMAYV) vaccinated mice was also used to look for antigenic cross-reactivity between CHIKV and MAYV structural proteins. After washing with PBS/T, membranes were incubated for 1 h with an HRP-conjugated goat anti-mouse IgG (Bio-Rad Cat. 170-6516). Finally, membranes were washed again using PBS/T and incubated with a chemiluminescent substrate (Clarity^TM^ Western ECL Blotting Substrates, BIO-RAD); signal was detected using a chemiluminescent Western blot imaging system (Image Lab, Bio-Rad).

### Charaterisation of purified VLPs by TEM

Formvar/carbon 200 Mesh Cu grids were glow-discharged in air and loaded with 3.5 μl of sample of CHIKV VLP. Excess liquid was removed, and the grids were washed 3 times with MilliQ water. Finally, grids were treated with 2% uranyl acetate for 30 s, excess uranyl acetate was carefully removed using filter paper. The grids were air-dried and analysed with a T12 transmission electron microscope (Thermo Fisher Scientific).

### Glycan analysis by western blotting

To determine the glycosylation of CHIKV VLPs and to understand viral biology and vaccine design, purified VLP sample was treated with PNGase F (New England Biolabs) and EndoF1-GST to determine their glycosylation status according to the protocol from NEB. Briefly, the samples were treated with 1 µl glycoprotein denaturing buffer (10X) in 9 µl of dH_2_O for 10 min at 95 °C. The denatured proteins were made into a total reaction volume of 20 µl by adding 2 µl GlycoBuffer 2 (10X), 2 µl 10% NP-40 buffer, 6 µl dH_2_O and 1 µl PNGase F or EndoH-GST and incubating for 1 h at 37°C. Treated and non-treated protein samples were analysed by SDS-PAGE and WB using sera from mice vaccinated with ChAdOx1 sCHIKV, ChAdOx1 sCHIKVΔC or anti-CHIKV E2 monoclonal antibody (Native Antigen Company, clone 18H01).

### Cryo-EM data collection and data processing

A 3.5-μl aliquot of purified VLP samples was applied to glow-discharged EM grids (UltraAuFoil, Quantifoil, or Lacey carbon grid, Ted Pella) and vitrified by plunge-freezing into liquid ethane using Vitrobot mark IV (Thermo Fisher Scientific) operated at 4 °C, 100% relative humidity. Cryo-EM data were acquired on a 300-kV Titan Krios (Thermo Fisher Scientific) equipped with an energy filter and a direct electron detector (K2 summit; Gatan) in counting mode. A total of 7856 movie stacks (19 frames) were collected in a total dose of 〜37 e^-^ Å^-2^ at 1.5–2.5 µm under focus. A nominal magnification of 70,000× was used, which resulted in a calibrated pixel size of 1.084 Å. Data processing was performed within the Scipion^83^. Movie frames were aligned and averaged with dose weighting in MotionCor2 to compensate for specimen drift^84^. The contrast transfer function (CTF) parameters were estimated using CTFFIND4^85^. Particles were picked from averaged images automatically using Xmipp3 auto-picking in ref. ^86^. Particles (24,103) were extracted in a box size of 800 pixels and 21,631 particles were finally retained after 2D and 3D classification for the subsequent 3D refinement in Relion 3.1^87^ with the icosahedral (I1) symmetry applied. After particle polishing and particle-based CTF refinement (PMID: 30713699), the final resolution was 5.7 Å based on the Fourier shell correlation at 0.143 criterion^88^. Localised reconstruction was performed to account for the flexibility of the E1-E2 layer^89,90^. Coordinates for subparticles centred at the trimers around icosahedral five-fold vertex (termed as trimer1) and at icosahedral three-fold symmetry axis (termed trimer2) were defined. A total of 1,030,383 and 407,400 subparticles were extracted for trimer 1 and trimer 2, respectively, using a box size of 256 pixels for 3D classification. 480,578 trimer1 subparticles and 177,563 trimer2 subparticles were selected out for separate 3D refinements, followed by refinement of the CTF parameters, resulting in the structures of trimer1 and trimer2 at 4.02-Å and 3.96-Å resolution. A composite cryo-EM map of CHIKV VLP was generated by combining trimer1 and trimer2 maps together in Scipion (protocol ‘localrec – stitch subparticles’).

### Glycopeptide analysis by nano LC-MS

CHIKV VLPs were proteolytically digested with trypsin (Promega) and chymotrypsin. Reaction mixtures were dried and peptides/glycopeptides were extracted using C18 Zip-tip (MerckMilipore) following the manufacturer’s protocol. Samples were resuspended in 0.1% formic acid prior to analysis by liquid chromatography-mass spectrometry using an Easy-nLC 1200 system coupled to an Orbitrap Fusion mass spectrometer (Thermo Fisher Scientific). Glycopeptides were separated using an EasySpray PepMap RSLC C18 column (75 μm × 75 cm) with a 240-min linear solvent gradient of 0–32% acetonitrile in 0.1% formic acid, followed by 35 min of 80% acetonitrile in 0.1% formic acid. Other settings include an LC flow rate of 200 nL/min, spray voltage of 2.8 kV, capillary temperature of 275 °C, and an HCD collision energy of 50%. Precursor and fragmentation detection were performed using an Orbitrap at the following resolution: MS1 = 100,000 and MS2 = 30,000. The automatic gain control (AGC) targets were MS1 = 4e5 and MS2 = 5e4, and injection times were MS1 = 50 and MS2 = 54. Glycopeptide fragmentation data were extracted from raw files using ByonicTM (Version 3.5.0) and ByologicTM (Version 3.5-15; Protein Metrics Inc.). Glycopeptide fragmentation data were manually verified. The extracted ion chromatographic areas for each true-positive glycopeptide, with the same amino-acid sequence, were compared to determine the relative quantitation of glycoforms at each specific N-linked glycan site.

### Production and purification of CHIKV E1/E2 antigens

The expression of CHIKVE1/E2 antigens was carried out in roller bottles using the standard PEI transfection of HEK293T cells according to the protocol previously described^82^.

### Mice and immunisation

Female inbred BALB/c (H-2d), (6–8 weeks) were used for the assessment of immunogenicity (*n* = 6 mice per group). Mice were purchased from Envigo. All animals and procedures were used in accordance with the terms of the UK Home Office Animals Act Project License. Procedures were approved by the University of Oxford Animal Care and Ethical Review Committee (PPL 30/2414). For induction of short-term anaesthesia, mice were anaesthetised using vaporised IsoFlo®. Mice were intramuscularly immunised with ChAdOx1 sCHIKV at a dose of 1 × 10^8^ IU as prime and boosted twice with Modified Vaccinia Ankara virus (MVA) encoding sCHIKV at a concentration of 1 × 10^7^ pfu per ml under isoflurane-induced anaesthesia as previously described^34,91^. All viral vector vaccines were prepared in endotoxin-free PBS and administered intramuscularly into right or left thigh muscle. All recombinant protein vaccinations were administered intramuscularly as a 50 µl dose containing sterile PBS and 3 µg (prime) or 10 µg (boost) of protein. For VLP vaccinations, 50 µl vaccine dose comprised PBS and 2 µg (prime) or 5 µg (boost) of VLP. All animals were humanely sacrificed at the end of each experiment by cervical dislocation.

### Enzyme-linked immunosorbent assay

Antibody binding to the recombinant CHIKV E1 or E2 antigens in mice serum after prime (P), prime-boost (P-B) and prime-boost-boost (P-B-B) immunisations was measured by an IgG enzyme linked immunosorbent assay (ELISA) as previous described^92–95^. Briefly, mice sera were diluted in Nunc Maxisorp Immuno ELISA plates coated with the CHIKVE1 or E2 diluted in PBS to a final concentration of 2 µg/mL and incubated at room temperature (RT) overnight. Plates were washed 6 times with PBST and blocked with 300 µL with Pierce^TM^ protein-free (PBS) blocking buffer (Thermo Fisher Scientific, Waltham, MA, U.S.) for 2 h at RT. Mouse serum was added and serially diluted 3-fold down in PBS/T with 50 µL per well as final volume and incubated for 2 h at RT. Following washing 6 times with PBS/T, bound antibodies were detected after a 1 h incubation with 50 µL of alkaline phosphatase-conjugated antibodies specific for whole mouse IgG (A3562-5ML, Sigma Aldrich, SLM, U.S.). Development was achieved using 100 µL of 4-nitrophenylphosphate diluted in diethanolamine buffer and the absorbance values at OD_405_ were measured and analysed using a CLARIOstar instrument (BMG Labtech, Aylesbury, GB). Serum antibody endpoint titres were defined by an absorbance value three standard deviations greater than the average OD_405_ of the control.

### Ex-vivo IFNγ ELISpot assay

ELISpots were carried out using peripheral blood mononuclear cells (PBMCs) using 20-mer specific CHIKV structural peptides overlapped by 10 a.a using previously described method^34^. Briefly, MAIP ELISpot plates (Millipore) were coated with an anti-mouse-IFN-γ mAb AN18 (Mabtech), after 1 h blocking with complete DMEM media. PBMCs were isolated from peripheral blood collected from tail vein bleeds into tubes containing 200 μl 10 mM EDTA/PBS. Erythrocytes were lysed using ACK lysis buffer and PBMCs harvested through centrifugation. Cells were washed, re-suspended in complete medium, counted using Countess Automated Cell Counter and 100 μl of media containing mouse PBMCs (at various concentrations) were plated into test wells. 50 μl of each of peptide pools diluted in media (10 μg/ml) and 5 × 10^6^ naïve splenocytes were added to test wells. Control wells with PBMCs from naïve mice without peptide and no cells with peptide were included. Plates were incubated at 37 °C, 5% CO_2_ for approximately 16 h. Plates were then washed with PBS and incubated with 50 μl biotinylated anti-mouse-IFN-γ mAb R4-6A2 (Mabtech) in PBS (1:1000) for 2 h. After washing, plates were incubated with a streptavidin alkaline phosphatase polymer (1:1000 in PBS) for 1 h. After another washing step, spots were developed by addition of colour development buffer (BIORAD). Once spots were visible, the reaction was stopped by washing the plate with water. Spots were counted using an ELISpot plate reader (AID, Germany) and expressed as spot-forming units (SFU) per million of PBMCs producing IFN-γ. Background responses in media-only wells were subtracted from those measured in wells stimulated by peptide pools.

### CHIKV VRP-based neutralisation assay

CHIKV neutralising antibody titres were determined as described previously using chimeric CHIKV replicon particles (VRPs) expressing Gaussia luciferase (Gluc) based on previously developed CHIKV VRP^69^. Briefly, 2 × 10^4^ BHK-21 cells were seeded in 96-well plates per well. The next day, VRPs (MOI of 5, calculated based on VRP RNA copies/ml) were preincubated with 2-fold serial dilutions of heat inactivated serum samples for 1 h at 37 °C before the mixtures were added to the 96-well plates. After incubation for 1 h at 37 °C, the inocula were removed, cells were washed with PBS, and medium was added. Readout of secreted Gaussia was performed at 24 h post infection using a Renilla luciferase assay system (Promega, Southhampton, U.K.). Sera-neutralisation-capacity was determined by measuring Gluc activity and relating it to Gluc readout after VRP application without serum. Results are presented as 50% neutralisation (NT50) titres. MAYV VRP-based neutralisation assay was used to determine the NT50 titres for MAYV as previously described^75^.

### CHIKV mouse challenge study

For the challenge study, female C57BL/6 J mice (4 weeks old) were obtained from Biotério Central do Instituto de Ciências Biomédicas (ICB) from São Paulo University (USP) as approved by the IACUC (6219031019). The virus strain CHIKV (strain LR2006) expressing the gene *Nanoluciferase* was kindly provided by Prof. Ana Carolina Gomes Jardim (Universidade Federal de Uberlândia). CHIKV was propagated and quantified by standard plaque assay in Vero CCL-81 cells as plaque forming units (PFU)^96^. C57BL/6 J mice (*n* = 6 per group) were immunised in 50 µl vaccine dose comprised of PBS and 5 µg (prime) and boosted with 5 µg of VLP at 2 weeks post-prime. CHIKV was inoculated subcutaneously in the ventral side of the right hind footpad toward the ankle with 5×10^6^ PFU CHIKV in 50 μl PBS. Viraemia was monitored daily from 3-day post-infection (dpi) until 10 dpi. Briefly, 10 μL of blood collected from the tail vein was diluted in 120 μL PBS and 10 μL citrate-phosphate-dextrose solution (Sigma-Aldrich). Viral RNA was extracted with E.Z.N.A. Viral RNA Kit (Omega Bio-Tek-R6874-02) in accordance with manufacturer’s protocol. To measure the levels of replicating virus in the extracted samples, RNA copies of negative sense *NsP*3 were quantified using RT-PCR (*Nsp3* Forward 5’- CGACGGATGCAGACGTGGTC -3’; Reverse 5’- ACATCGCAGTCTATGGAGATGTGC -3’; probe 5’-VIC TGCGGACCCAAGTGGAGCTGCTGGA BHQ-1-3’). The reaction was normalised using Quant Studio 3 Real-Time PCR System (Thermo Fisher Scientific) and the standard curve was generated from the viral stock previously titled. Joint swelling of the footpad was scored starting at day 0 and after from 3 to 10 dpi. Using a digital caliper, the hind paw swelling outcome was done for both height (thickness) and breadth of the foot and were quantified as [height x breadth]. All animals were humanely sacrificed at the end of each experiment by cervical dislocation.

## Data Availability

All data generated and analysed in this study are included in the manuscript or are available from the authors upon request. The cryo-EM maps of CHIKV VLP icosahedral reconstruction, in addition to the localised reconstructions of trimer 1 and trimer 2, and the composite map of the CHIKV VLP, have been deposited in the Electron Microscopy Data Bank (EMDB) under the accession codes EMD-54410, EMD-54411, EMD-54412 and EMD-54413, respectively.
